# Acarbose improves health and lifespan in aging HET3 mice

**DOI:** 10.1111/acel.12898

**Published:** 2019-01-27

**Authors:** David E. Harrison, Randy Strong, Silvestre Alavez, Clinton Michael Astle, John DiGiovanni, Elizabeth Fernandez, Kevin Flurkey, Michael Garratt, Jonathan A. L. Gelfond, Martin A. Javors, Moshe Levi, Gordon J. Lithgow, Francesca Macchiarini, James F. Nelson, Stacey J. Sukoff Rizzo, Thomas J. Slaga, Tim Stearns, John Erby Wilkinson, Richard A. Miller

**Affiliations:** ^1^ The Jackson Laboratory Bar Harbor Maine; ^2^ Barshop Institute for Longevity and Aging Studies The University of Texas Health Science Center at San Antonio San Antonio Texas; ^3^ Geriatric Research, Education and Clinical Center South Texas Veterans Health Care System San Antonio Texas; ^4^ Research Service South Texas Veterans Health Care System San Antonio Texas; ^5^ Department of Pharmacology The University of Texas Health Science Center at San Antonio San Antonio Texas; ^6^ Buck Institute for Research on Aging Novato California; ^7^ Metropolitan Autonomous University Lerma Mexico; ^8^ University of Texas at Austin Austin Texas; ^9^ Department of Pathology University of Michigan Ann Arbor Michigan; ^10^ Geriatrics Center University of Michigan Ann Arbor Michigan; ^11^ Department of Epidemiology & Biostatistics The University of Texas Health Science Center at San Antonio San Antonio Texas; ^12^ Department of Psychiatry The University of Texas Health Science Center at San Antonio San Antonio Texas; ^13^ Georgetown University Washington District of Columbia; ^14^ Division of Aging Biology National Institute on Aging Bethesda Maryland; ^15^ Department of Physiology The University of Texas Health Science Center at San Antonio San Antonio Texas; ^16^ Unit for Laboratory Animal Medicine and Department of Pathology University of Michigan Ann Arbor Michigan

**Keywords:** acarbose, health measures, heterogeneous mice, lifespan

## Abstract

To follow‐up on our previous report that acarbose (ACA), a drug that blocks postprandial glucose spikes, increases mouse lifespan, we studied ACA at three doses: 400, 1,000 (the original dose), and 2,500 ppm, using genetically heterogeneous mice at three sites. Each dose led to a significant change (by log‐rank test) in both sexes, with larger effects in males, consistent with the original report. There were no significant differences among the three doses. The two higher doses produced 16% or 17% increases in median longevity of males, but only 4% or 5% increases in females. Age at the 90th percentile was increased significantly (8%–11%) in males at each dose, but was significantly increased (3%) in females only at 1,000 ppm. The sex effect on longevity is not explained simply by weight or fat mass, which were reduced by ACA more in females than in males. ACA at 1,000 ppm reduced lung tumors in males, diminished liver degeneration in both sexes and glomerulosclerosis in females, reduced blood glucose responses to refeeding in males, and improved rotarod performance in aging females, but not males. Three other interventions were also tested: ursolic acid, 2‐(2‐hydroxyphenyl) benzothiazole (HBX), and INT‐767; none of these affected lifespan at the doses tested. The acarbose results confirm and extend our original report, prompt further attention to the effects of transient periods of high blood glucose on aging and the diseases of aging, including cancer, and should motivate studies of acarbose and other glucose‐control drugs in humans.

## 1 INTRODUCTION

The ITP finds interventions that improve mammalian lifespan, and the current study determines optimal doses of ACA. These studies suggest new insights into the factors that modulate aging rates and may lead to treatments useful in the clinic. Such interventions also suggest hypotheses for basic research, as different biological systems are compared, and different models of delayed aging contrasted to distinguish changes essential in delaying aging.

The ITP design, presented at this URL: (http://www.nia.nih.gov/research/dab/interventions-testing-program-itp), uses genetically heterogeneous (UM‐HET3) mice, the offspring of the CByB6F1 × C3D2F1 cross. Such crosses of two diverse F1 hybrids, representing four different inbred strains, produce genetically heterogeneous populations in which each animal is unique, but a full sibling of all other mice in the population, so that the cross is reproducible (Roderick, 1963). Use of genetically diverse individuals minimizes the possibility that characteristics of a single inbred or F1 hybrid genotype might be confused with those of the species. The ITP includes replication at three sites, with sufficient mice to give more than 80% power to detect a change of 10% in mean lifespan, even if only two of the three sites contribute data. This design was detailed previously (Harrison et al., 2014, 2009; Miller et al, 2011, 2014; Strong et al., 2016, 2013, 2008).

The interventions for this study were chosen for the following reasons:

1. Acarbose (ACA) is a candidate to replicate some aspects of diet or caloric restriction. Archer (2003) suggests that the postmeal spike in glucose may contribute to aging. This spike is reduced by ACA (Balfour & McTavish, 1993). Acarbose has been widely used clinically to prevent postprandial hyperglycemia, and several reports (Frantz et al., 2005; Miyamura et al., 2010) demonstrate its ability to reduce or prevent postprandial hyperglycemia in mice. The glucose spike during a meal is blunted because acarbose inhibits α‐glucosidase, thus reducing the rate at which polysaccharides are digested, as well as reducing sugar uptake. It does not sequester glucose, nor block its uptake—it blocks its release from complex polysaccharides.

Like chronic diet restriction (DR), chronic ACA treatment reduces body weight and body fat, and also improves glucose dysregulation associated with aging (Yamamoto & Otsuki 2006). However, unlike DR, food intake is often increased, not reduced, during long‐term ACA treatment (Yamamoto & Otsuki 2006). Importantly, ACA increases HET3 mouse lifespans more effectively in males than in females (Harrison et al., 2014; Strong et al., 2016), while DR increases lifespan to a similar degree in both (Flurkey et al., 2010).

Once a longevity study has shown a benefit at the drug dose tested initially, it is very useful to evaluate the same drug over a wider range of doses to try to determine the optimal dose for beneficial effect. Lower doses could, in principle, be more effective than the original dose, if the lower dose mitigates harmful side effects. Doses above the one originally tested could, in principle, be more effective, if benefits are proportional to drug concentrations over the tested range. A drug that led to sex‐specific benefits at the original dose may, in principle, show strong effects in both sexes, if, for example, its blood or tissue concentration is affected by sex‐specific metabolic pathways. Better definition of optimal drug dose is also very helpful in providing a foundation for further studies. For these reasons, three different doses of ACA are tested here.

- Ursolic acid (UA) decreased d‐galactose (D‐gal)‐induced neurotoxicity in mice (Lu et al., 2007) and also inhibited cognitive impairment induced by a high‐fat diet (Lu et al., 2011). Kunkel et al. (2012) found that UA increased skeletal muscle mass and function while improving glucose tolerance and reducing obesity. These results suggest that UA might benefit deleterious effects of age.

- 2‐(2‐hydroxyphenyl) benzothiazole (HBX) is a compound with similar structural features to the flavonoid Thioflavin T (4‐(3,6‐dimethyl‐1,3‐benzothiazol‐3‐ium‐2‐yl)‐N,N‐dimethylaniline chloride), a molecule used in histopathology to stain amyloid in tissues. These molecules are able to maintain protein homeostasis during aging, and to increase median and maximal lifespans in *C. elegans* (Alavez et al., 2011).

- INT‐767d (6α‐ethyl‐24‐nor‐5β‐cholane‐3α,7α,23‐triol‐23 sulfate sodium salt) is a dual FXR/TGR5 activating agonist. Activating the bile acid‐activated nuclear hormone receptor FXR, and the G protein coupled receptor TGR5, reduces several diseases of aging, including chronic liver and kidney disease as well as diabetes (Rizzo et al., 2010). Chronic treatment of aging mice with INT‐767d could, in principle, retard these diseases as well as other deleterious aspects of aging (Fiorucci, Mencarelli, Palladino, & Cipriani, 2009; Hylemon et al., 2009; Wang et al, 2017). Dwarf mouse models with increased lifespan also have increased serum and liver bile acid levels and FXR activation, which supports this idea (Gems, 2007).

## 2 RESULTS

### 2.1 Lifespan and body weight

**Figure 1:**
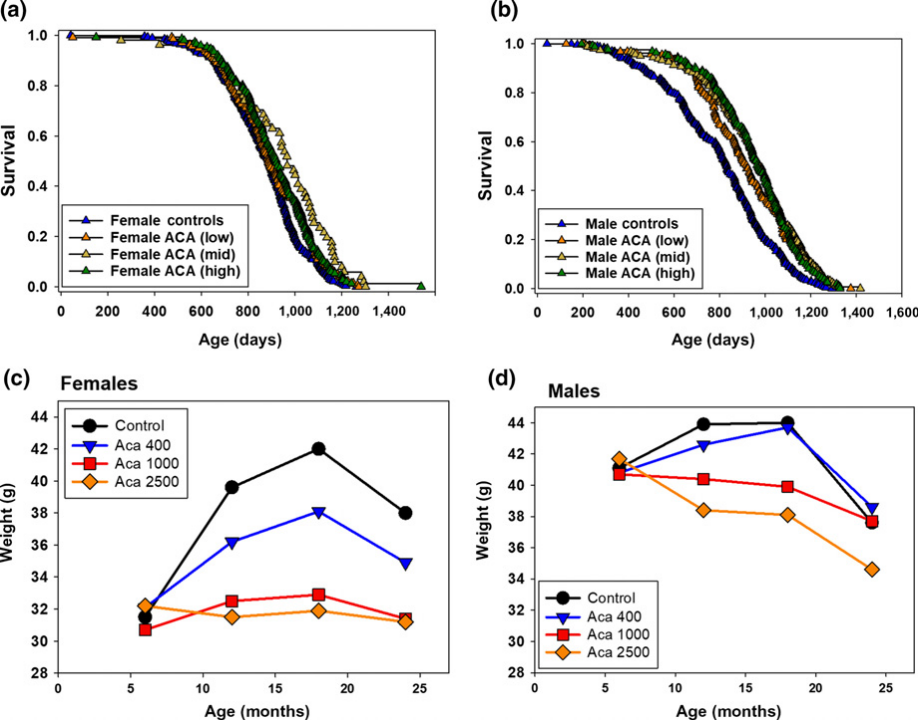
Effects of ACA on lifespan and body weight. Effects of dietary ACA dose on: (a, b) lifespans; (c, d) body weights over lives of the same mice. Lifespan curves (a and b for females and males) show the entire lifespan from the data used to produce Table 1, which includes sample sizes and statistical analyses of differences in median and 90th percentile values from the control as a result of each ACA dose. Body weights (c and d for females and males) represent 115–165 mice tested at 6, 12, 18, and 24 months of age in the ACA‐fed groups. Numbers of controls were about twice as high. Using one‐way ANOVA with Sidak post hoc test, there was no effect of ACA at 6 months. At 12 and 18 months, weights were significantly different as shown by >: Males – Control, ACA_lo > ACA_hi, ACA_mid; Females – Control > ACA_lo > ACA_mid, ACA_hi. At 24 months of age, there were no significant differences between Control and treated males, while in Females – Control, ACA_Lo > ACA_hi, ACA_mid. Weights at 24 months are hard to interpret, due to unbalanced death rates at the three sites, and possible weight loss due to ill health

Mice were fed ACA at three different doses: low—400, medium—1,000, and high—2,500 mg per kg diet (ppm) from 8 months of age. Using data pooled across the three sites, ACA had significant effects by the log‐rank test on female median lifespans at all three doses. At medium and high doses, median lifespan increased 5% (*p* = 0.003) and 4% (*p* = 0.006), respectively. The lowest dose of ACA did not change median survival (0% increase), but significantly (*p* = 0.03) improved survival at ages greater than the median (Table 1, Figure 1a). ACA had much more benefit in males, whose median lifespans were increased by the three doses by 11%, 17%, and 16%, respectively, all highly (*p* < 0.0001) significant (Table 1, Figure 1b). None of the effects on lifespan of the three ACA doses were significantly different from the other two doses in either males or females. We used the 90th percentile as a surrogate for maximum lifespan, and the Wang/Allison test (Wang, Li, Redden, Weindruch, & Allison, 2004) in pooled data also showed much larger benefits in males. The low, medium, and high doses of ACA caused increases of 2%, 3%, and 3% (*p* = 0.37, 0.007, 0.10), respectively, in females, and 11%, 11%, and 8% (*p* = 0.0004, 0.0004, 0.0001), respectively, in males (Table 1). Neither UA, I767 nor HBX changed lifespan significantly in either males or females at the doses used.

**Table 1:** Effects of three different doses of ACA on lifespan

Group	*N*	Median lifespan			Lifespan at 90th percentile		
		Days	% change	*p*‐value	Days	% change	Wang–Allison *p*‐value
Females							
Cont_13	287	889			1,097		
ACA_lo	139	887	0%	0.03	1,123	2%	0.37
ACA_mid	142	933	5%	0.003	1,125	3%	0.007
ACA_hi	152	922	4%	0.006	1,125	3%	0.10
UA	147	885	0%	0.49	1,084	–1%	0.46
Cont_12	279	877			1,100		
HBX	136	856	–2%	0.4	1,091	–1%	NS
I767d	136	868	–1%	0.8	1,102	0%	NS
Males							
Cont_13	273	830			1,089		
ACA_lo	147	918	11%	<0.0001	1,211	11%	0.0004
ACA_mid	161	975	17%	<0.0001	1,210	11%	0.0004
ACA_hi	163	964	16%	<0.0001	1,181	8%	0.0001
UA	151	883	6%	0.38	1,092	0%	0.91
Cont_12	284	816			1,055		
HBX	155	822	1%	0.4	1,069	1%	NS
I767d	147	791	–3%	0.11	1,012	–4%	NS

Note

Lifespans of ITP mice from cohorts 2013 and 2012. Cont_13 = controls for cohort started in 2013; Cont_12 = controls for cohort started in 2012. *N* = number of mice tested; data were pooled, with about 1/3 from each testing site. Probabilities that lifespans are the same as the controls in column 4 (*p*‐value) used two‐tailed log‐rank test on pooled data stratified by sites; “removed” mice were included in these calculations. Probabilities that the proportion of live mice is the same in treated as in the control group at the 90th percentile age are evaluated by the procedure of Wang et al., (2004). Amounts of ACA in diet: hi = 2,500 ppm; mid = 1,000 ppm; lo = 400 ppm. UA, HBX, and 1767d had no significant effect on lifespan in the doses used.

NS: not significant.

Site differences showed the importance of replication (Table 2) and the value of pooling data to increase sample size and statistical power. Effects of ACA were not significant in females at either TJL or UM, although they were at UT ranging from *p* = 0.02 to 0.03. In males, all three doses of ACA increased lifespan significantly at TJL and UT (*p* = 0.002 to <0.0001); only the 1,000 ppm dose gave a significant increase in male lifespan at UM (*p* = 0.006). Percentage increases in lifespan—given for diets containing 400, 1,000, and 2,500 ppm ACA, respectively, in Table 2—also were larger at TJL (10, 20, 20) and UT (14, 13, 19) than at UM (1, 13, 7), perhaps reflecting the 14% longer lifespan of control males at the UM site. As in past ITP cohorts, lifespan for control females was similar at all three sites (median values at TJL—890 days, UM—870 days, and UT—897 days), but longer for males at UM (TJL—803 days, UM—912 days, and UT—807 days).

**Table 2:** Effects of ACA on median lifespan at each ITP site

Group	TJL			UM			UT			Mean % change
	Days	% change	p‐value	Days	% change	p‐value	Days	% change	p‐value	
Females										
Cont_13	890			870			897			
ACA_lo	887	0%	0.43	871	0%	0.49	923	3%	0.03	1%
ACA_mid	934	4%	0.33	890	1%	0.06	950	4%	0.02	3%
ACA_hi	938	6%	0.13	931	6%	0.32	917	2%	0.03	5%
Males										
Cont_13	803			912			807			
ACA_lo	880	10%	0.001	924	1%	0.21	919	14%	0.0006	8%
ACA_mid	967	20%	0.000	1,033	13%	0.006	914	13%	0.002	16%
ACA_hi	960	20%	0.000	975	7%	0.21	957	19%	0.000	15%

Note

For each site, this table lists median lifespans, % change, and the log‐rank *p*‐value from the control (Cont_13) for mice fed the three different doses of ACA starting at 8 months of age. A *p*‐value of 0.000 means *p* < 0.001. The rightmost column shows averages (“Mean % change”) of median changes across the three sites.

**Table 3:** Effects of ACA on weight and body composition at TJL

Group	Body weight (g)	Fat weight (g)	Lean weight (g)	Body weight (g)	Fat weight (g)	Lean weight (g)	Body weight (g)	Fat weight (g)	Lean weight (g)
Females	17 months			23 months			29–30 months		
Control	44 ± 2	17 ± 2	21 ± 1	43 ± 3	15 ± 3	21 ± 1	37 ± 3	8 ± 3	23 ± 1
ACA	33 ± 2**	7 ± 1**	21 ± 1	36 ± 3	8 ± 2*	21 ± 2	29 ± 2*	2 ± 1*	20 ± 2*
Males	16 months			22–23 months			29–30 months		
Control	42 ± 2	8 ± 2	26 ± 3	42 ± 4	9 ± 3	21 ± 1	34 ± 3	3 ± 2	24 ± 2
ACA	42 ± 1	7 ± 1	25 ± 2	41 ± 2	7 ± 2	21 ± 2	35 ± 1	2 ± 1	27 ± 1

Note

Mice were tested using the EchoMRI™ 3‐in‐1. The ages of the mice are given for each sex. ACA‐treated mice were fed ACA at 1,000 ppm starting at 4 months of age. The mice tested were not the same mice used for lifespan studies. Statistical tests were a two‐way repeated measures ANOVA. Numbers of HET3 mice tested at 16–17, 22–23 and 28–29 months—Females: ACA‐treated (12, 9, 4); Controls (14, 13, 5). Males: ACA‐treated (20, 18, 14); Controls (9, 4, 4).

** Control differs from ACA‐fed *p* < 0.0001.

* Control differs from ACA‐fed *p* = 0.04 to 0.01.

**Figure 2:**
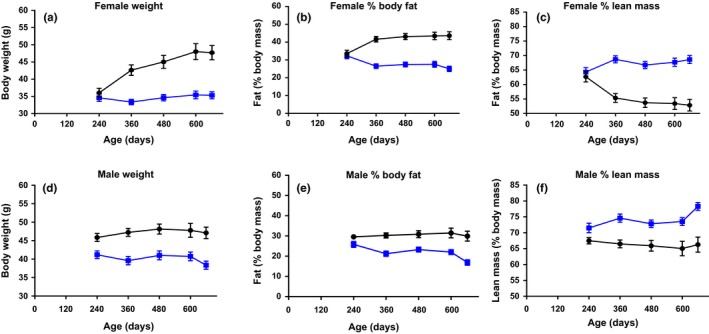
Changes with age in body composition due to dietary ACA. Effects of 1,000 ppm ACA diet starting at 8 months of age on weights and body composition from 8 to 22 months of age at UT. Giving numbers of mice at 8–22 months of age: females (32–28 controls, 32–28 ACA‐treated); males (32–14 controls, 33–30 ACA‐treated). The same groups of mice were tested at 8, 12, 16, 20, and 22 months. ACA‐treated have blue dots; controls black dots. Data are shown as mean ± *SE*, and weights and body fat were significantly lower, while lean mass was significantly higher in both males and females fed the ACA diet as measured by 2‐way ANOVA using GraphPad version 7.03, which accounted for the missing mice

**Figure 3:**
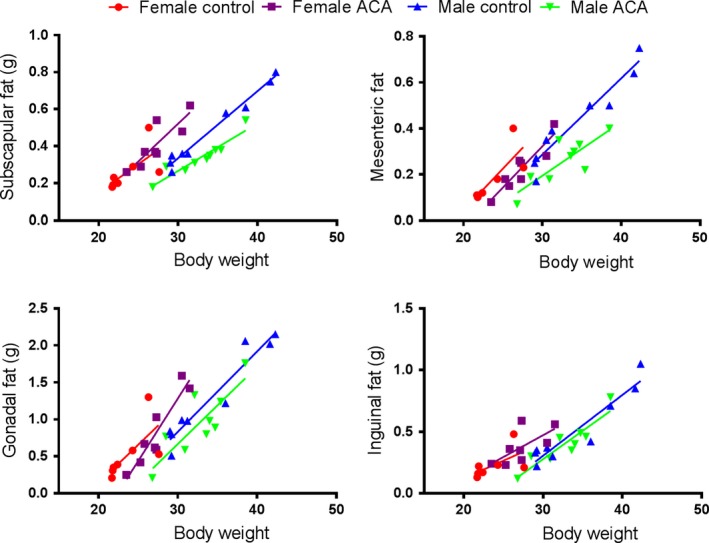
Effects of ACA on fat depends on specific fat type. Effects of ACA on amount of fat relative to body weight in 12‐month‐old HET3 mice at UM. Numbers of male ACA and controls are 9 each, while 8 females received ACA and 6 were controls. In subscapular fat, ACA effects are sex‐specific: females, *p* = 0.71; males, *p* < 0.001. In mesenteric fat, ACA results in a strong reduction in both sexes: *p* < 0.001. In gonadal fat, ACA effects are similar in both sexes, but weak: *p* = 0.074. In Inguinal fat, ACA has no effect. *p* values, from analysis of covariance, reflect differences in the intercept term, which measures whether ACA alters fat pad weight after adjustment for body weight. HET3 mice were fed diet with 1,000 ppm ACA starting at 4 months; controls were fed the base diet. Mice were fasted for 18 hr prior to dissection. This was part of a larger study in which mice had a sham‐operation procedure at age 3 months. They were anesthetized, gonads were exteriorized through an incision and then returned to the abdominal cavity, and the wound closed

**Figure 4:**
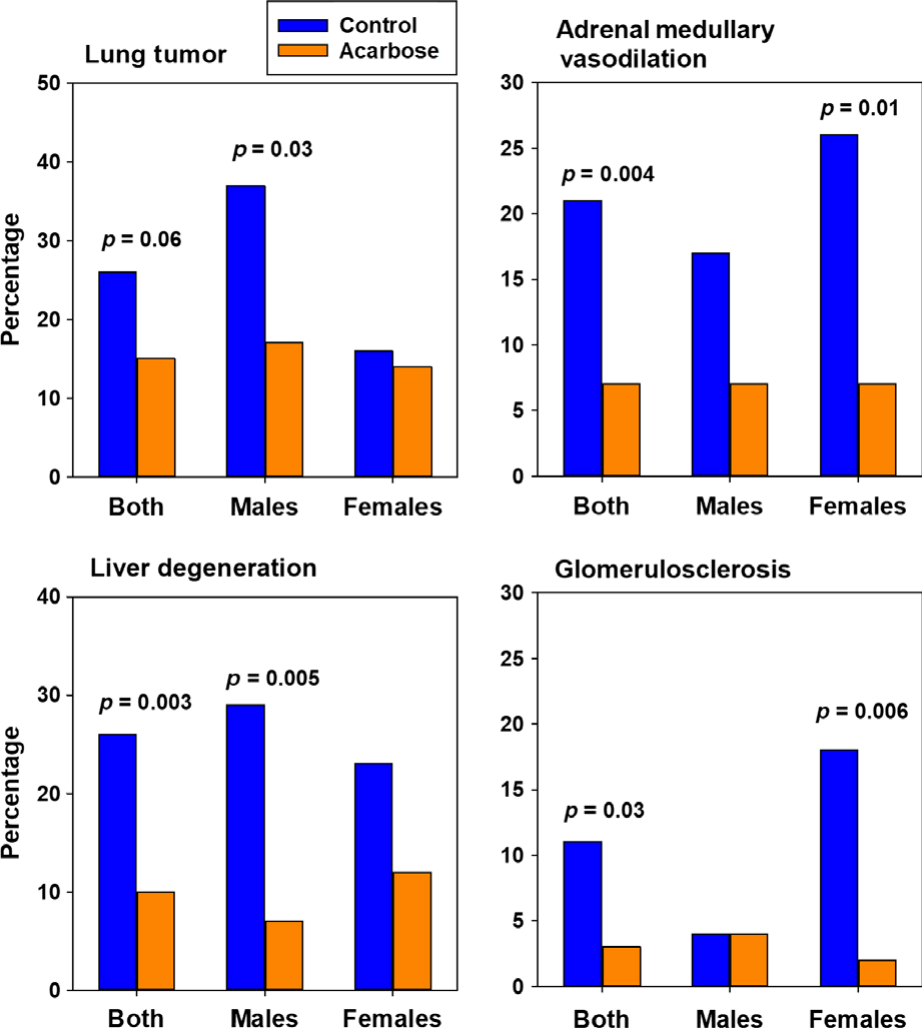
Microhistopathology effects of ACA. Lesions are compared in controls and mice fed the 1,000 ppm ACA diet starting at 4 months of age. Numbers of mice: ACA old – 57 F and 54 M. The Control group consisted of 41 male and 43 female animals, of which 11 males and 14 females were contemporaneous with the current ACA population, and the remainder consisted (as a preplanned strategy) of controls from earlier cohorts also evaluated at 22 months of age. About a third of the mice came from each site. The values shown are percentage of cases with the indicated lesions, and p‐values reflect differences between two proportions using an asymptotically, normally distributed *z* statistic, as documented in the STATA program

Using pooled data from the same mice, when fed 1,000 or 2,500 ppm ACA, body weights averaged 4–6 g lower in females at 12, 18, and 24 months of age (Figure 1c) and 2–4 g lower in males at 12 and 18 months of age (Figure 1d). Statistically, female controls were the heaviest at 12 and 18 months, with females fed 400 ppm ACA next most heavy. Those fed 1,000 or 2,500 ppm ACA were the lightest, with similar body weights (Figure 1c). At 12 and 18 months, male controls and those fed 400 ppm ACA were the most heavy, while those fed 1,000 or 2,500 ppm ACA were lighter, and not significantly different from each other (Figure 1d).

### 2.2 Body composition

Acarbose at 1,000 ppm was fed to separate groups of mice starting at 4 months of age, and weights and body composition then evaluated at two sites. At TJL and UT, fat and lean weights were measured by NMR body composition analysis, using the echo MRI^TM^ 3‐in‐1 (Table 2), in which grams of fat are the mass of the body's fat molecules expressed as g of canola oil, while grams of lean are muscle tissue mass equivalent of all body parts containing water, excluding fat, bone, and substances that do not contribute to the NMR signal, such as hair, teeth, and claws. Testing the same groups of ACA‐fed and age‐ and sex‐matched control mice at 16–17, 22–23 and 29–30 months of age, ACA reduced body and fat weights significantly in females, but had no significant effects on these measures in males. Lean mass was not affected by ACA in either sex (Table 2). At UT, ACA diets also had greater effects in females, reducing body weights by 8–12 g in females at 12–22 months of age (Figure 2a), and by 6–8 g in males over the same age range (Figure 2d). The decrease in % female body fat (Figure 2b) was about twice as great as in males (Figure 2e), while the increase in % lean mass in females fed ACA (Figure 3c) was two to three times greater than in males (Figure 2f).

A separate set of data from UM defined the effects of ACA feeding on four fat depots using HET3 mice at 12 months of age (Figure 3). In subscapular fat, there were no ACA effects in females, but highly significant effects in males (*p* < 0.001). In mesenteric fat, ACA caused highly significant reductions in both sexes (both *p* < 0.001). In gonadal fat, ACA effects were also similar in both sexes, but did not reach statistical significance, and in inguinal fat, ACA had no effect (Figure 3).

### 2.3 Pathology

As illustrated in Figure 4, males fed ACA had about half as many lung tumors as controls, but ACA feeding did not reduce the already low frequency of lung tumors in females. The frequency of adrenal medullary vasodilation was reduced to an equivalent extent in both sexes, reaching statistical significance in females, and in the pooled data, though not in males (Figure 4). Replicating results reported by Strong et al. (2016), ACA reduced the incidence of liver degeneration in both male and female UM‐HET3 mice, but only results in males and the pooled population were significant. Finally, females fed ACA had less glomerulosclerosis, but not males in which glomerulosclerosis was rare even in controls (Figure 4). Lesions that were not affected by ACA feeding, to a significant degree, included endometrial hyperplasia, lymphoma, pancreatic atrophy, renal mineralization, adrenal hyperplasia, adrenal cortical degeneration, hepatic microgranuloma, uterine cysts, uterine cystic endometrial hyperplasia, ovarian atrophy, and ovarian pigment/lipofuscin; inferences about these negative findings would require confirmation using a larger number of test cases.

### 2.4 Blood glucose after refeeding

**Figure 5:**
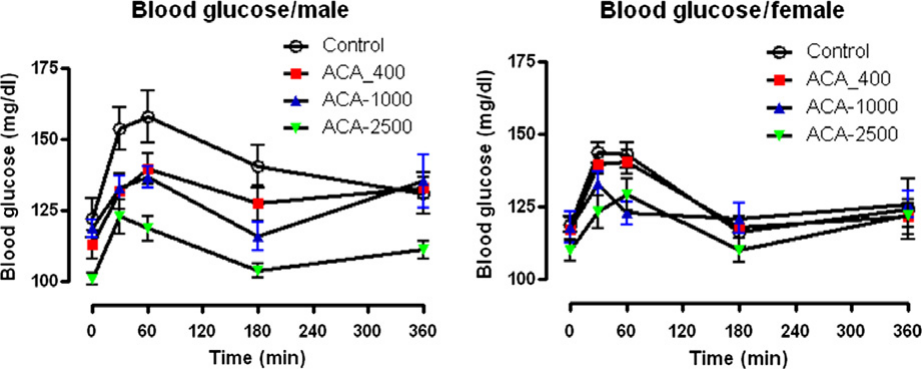
ACA effects on blood glucose after refeeding. Postprandial blood glucose is reduced in males but not in females by 6 weeks on ACA diets. Male and female UM‐HET3 mice were given one of four diets for 6 weeks starting at 4 months of age. Each point represents the mean ± *SEM* of 10 mice, tested at the indicated times. Green symbols indicate diets formulated with ACA at 2,500 ppm; blue 1,000 ppm; red 400 ppm; and white 0 ppm (control). Mice were fasted from 18:00 until 9:00 the next day. Blood glucose was measured at “0” min (before food was returned), and at 30, 60, 180, and 360 min after the food was returned to their cages. All measures were made in each sex in a single session, but in different sessions for each sex. Data were analyzed using RM one‐way analysis of variance (ANOVA) for male and female mice separately. In both sexes, we compared the differences between the four groups using GraphPad Prism 7.03. In females, the different diets did not affect blood glucose levels significantly (*p* = 0.092). In males, the different diets had significant effects on blood glucose (*p* = 0.002)

Figure 5 shows young (4 month old) males and females that were fed either control diets (0 ppm ACA) or diets containing 400, 1,000, or 2,500 ppm ACA for 6 weeks. Food was removed at 6 p.m., and returned at 9 a.m. the next day. Blood glucose levels were measured just before food was returned, and 30, 60, 180, and 360 min after it was returned. There were no significant differences in females, but highly significant (*p* < 0.002) differences in males, in which higher levels of ACA in diets reduced blood glucose after refeeding.

### 2.5 Rotarod performance with training

**Figure 6:**
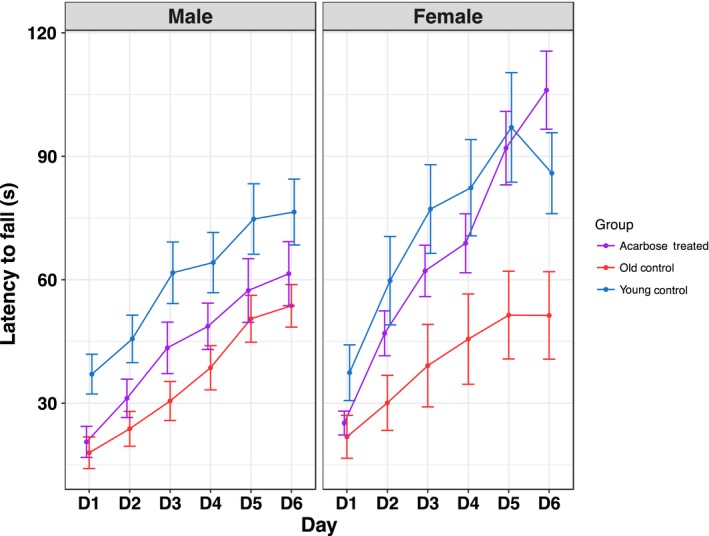
Effects of ACA on rotarod performance. Training on a rotarod was more effective in females fed ACA, but there was no benefit in males. Groups of male and female UM‐HET3 mice were fed control or acarbose‐containing diets (1,000 ppm) beginning at 8 months of age until they were 22 months of age. A group of 4‐month‐old mice fed the control diet served as the young control group. Mice were trained on a rotarod for 5 days, with a final test done on day 6, and the latency to fall was tested. Average performance is shown by treatment, day, and sex. Error bars represent standard errors of the means. Sample sizes for female mice were as follows: young control = 19, ACA‐fed = 27, and old control = 27; and for male mice: young control = 19, ACA‐fed = 29, and old control = 14. Since mice with lower weights tended to have higher rotarod times, weight was regressed onto group, and the residuals from this model used to adjust for weight in an ANCOVA comparing averages between groups (young vs. acarbose vs. untreated)

Rotarod testing was conducted, and average performance by sex, group, and day is shown in Figure 6. On test day 6, after 5 days of training, ACA females performed better than age‐matched old controls (*p* = 0.0001), while male mice did not show significant benefits. Young female controls performed better than old controls (*p* = 0.02), while untreated males showed the same trend but it was not statistically significant (*p* = 0.06). In a second measure of performance—mean latency to fall averaged over the 6‐days—female mice fed ACA again performed better than age‐matched controls (*p* = 0.02), and young female controls performed better than old controls (*p* = 0.006). Again, ACA did not lead to significant improvement in male mice, although young male controls performed better than old (*p* = 0.007). In a measure of learning rate, the rate of change across the 6‐day training period, ACA‐fed females again performed better than age‐matched controls (*p* = 0.009), and again, old female controls performed less well than young (*p* = 0.01). Male learning rates did not differ among younger controls, ACA‐treated, or old controls (all *p* > 0.05).

## 3 DISCUSSION

### 3.1 Effects of ACA on lifespan, weight, and body composition

Key findings are that ACA improves male lifespan over a broad range, with significant effects from diets containing 400, 1,000, or 2,500 ppm ACA. At all three doses, ACA extended lifespan much more strongly in males than in females (Tables 1 and 2), offering an interesting model for sex differences. In Figure 5, ACA affects daily postprandial increases in glucose much more in males than in females, so males may be more amenable to metabolic benefits from ACA. Most UM‐HET3 mice die with some form of cancer, so ACA effects on overall lifespan probably reflect protection against neoplastic disease.

As in previous ITP cohorts (Harrison et al., 2014, 2009; Miller et al., 2011, 2014; Strong et al., 2016, 2008), unknown site‐specific differences led to male control mice living longer at UM than at UT and TJL. Female controls in the same cohorts had very similar lifespans at all three sites. This offers opportunities to explore sex‐specific aspects of aging.

If the longevity benefit of ACA were due strictly to its effects on weight and fat, one might expect that its lifespan benefit would be stronger in females than in males, contrary to our findings. Weights were reduced more in females than in males (Figure 1c,d), as were amounts of fat (Table 2, Figure 2). Thus, the lengthened survival for ACA‐treated males vs. ACA‐treated females cannot be explained solely by changes in body weight or fat, suggesting that the ACA benefit on lifespan is not directly due to the effects of diet restriction (DR), which increases male and female lifespans to a similar degree in HET3 mice (Flurkey et al., 2010). Furthermore, ACA and DR have opposite effects on blood levels of FGF21 and on voluntary activity (Harrison et al., 2014), showing that these treatments differ. Effects of ACA and DR may differ in HET3 mice due to carbohydrate vs. total diet restriction. Differences could reflect differences in the microbiome (Smith et al., 2018) caused by the different treatments, or factors still to be elucidated.

Besides ACA, significant increases in male lifespans are caused by 17‐α‐estradiol (17aE2), aspirin, Protandim, and nordihydroguaiaretic acid (NDGA) (Harrison et al, 2014; Strong et al., 2016, 2008) and by rapamycin in both male and female mice (Harrison et al., 2014, 2009; Miller et al., 2011, 2014; Wilkinson et al., 2012). Rapamycin also improves type II diabetic models (Reifsnyder et al., 2015, 2016, 2017) and brain histone modifications (Gong et al., 2015). Effects with 17aE2 may be explained by metabolomic responses modulated by gonadal hormones (Garratt, Tsai, Galecki, Jain, & Miller, 2018).

### 3.2 Specific physiological effects of ACA

Further key findings are from detailed studies of ACA‐treated and control mice at each site. At UM, effects of ACA on amount of fat relative to body weight in 12‐month‐old HET3 mice differ greatly depending on which fat type is examined (Figure 3). In only one fat type, subscapular, are ACA effects sex‐specific, with no effects in females and a highly significant reduction of fat relative to body weight in males. Perhaps subscapular fat might be important in predicting effects of drugs on lifespan, although tests at a wider variety of ages and with a wider variety of interventions are essential before concluding this is the case.

Figure 4 presents pathology of mice, 22–25 months of age, which had been fed 1,000 ppm ACA (57 F and 54 M) and three pooled groups of age‐matched controls (43 F and 41 M). HET3 mice show a wide range of lesions, making them a good model for normal populations, but the sparse distribution of many specific lesions among 22‐ to 25‐month‐old HET3 mice limits statistical power for specific pathologies. Nevertheless, 37% of male controls had lung tumors, and ACA reduced this by half, which might explain some of the increase in male lifespans.

In fasting and refeeding assessed at UT (Figure 5), glucose levels were not affected by ACA in females, but were effects in males were highly significant, and higher amounts of ACA led to lower levels of blood glucose. This test was done using mice at 5.5 months of age, which had been fed ACA for only 6 weeks. While it suggests a reason for larger effects on male lifespans, effects of ACA should be tested over longer periods of time. Lamming et al. (2013) showed no significant decrease of insulin sensitivity or increase in glucose with age in either male or female HET3 mice, but these were average numbers taken during the day when mice were not eating; it is possible that postprandial surges in blood glucose may have effects on health and lifespan independent of mean levels of glucose. Hormones of the gonadal–pituitary axis may be important in sex differences. Yuan et al. (2012) showed that female sexual maturation co‐regulated with lifespan via IGF1, while Garratt, Bower, Garcia, and Miller (2017) showed that gonadal hormones were important in differences between males and females on several effects of ACA, including glucose homeostasis, although endocrine effects on lifespan were not evaluated. Benefits of ACA on rotarod performance (Jones & Roberts, 1968) are highly significant in females but not in males (Figure 6). These data suggest that lifespan measures do not detect the advantage given by ACA to females in the agility tested by the rotarod.

The National Institute on Aging Interventions Testing Program (ITP) has previously reported significant increases in lifespan caused by aspirin, Protandim, and nordihydroguaiaretic acid (NDGA) in male mice (Strong et al., 2016, 2008) and by rapamycin in both male and female mice (Harrison et al., 2014, 2009; Miller et al., 2011, 2014; Wilkinson et al., 2012). Rapamycin also improves type II diabetic models (Reifsnyder et al., 2015, 2016, 2017) and brain histone modifications (Gong et al., 2015). Acarbose and 17‐α‐estradiol (17aE2) also extend mouse lifespans, with stronger (ACA) or exclusive (17aE2) effects in males (Harrison et al., 2014; Strong et al., 2016). Effects with 17aE2 may be explained by metabolomic responses modulated by gonadal hormones (Garratt et al., 2018).

The lifespan studies of the ITP show that adhering to rigorous standards provides strong evidence that mouse lifespan can be reproducibly extended by drugs in the diet (Harrison et al., 2014, 2009; Miller et al., 2011, 2014; Strong et al., 2016, 2008; Wilkinson et al., 2012). The growing arsenal of drugs that extend lifespan, perhaps by modulation of aging, cancer, or both, provides raw material for mechanistic studies. It also will complement work done using mutant stocks and dietary interventions to delineate the factors that control aging rate in mammals and link aging to multiple forms of illness.

## 4 EXPERIMENTAL PROCEDURES

### 4.1 Mouse production, maintenance, and estimation of lifespan

UM‐HET3 mice were produced at each of the three test sites as previously described (Harrison et al., 2009; Miller et al., 2011; Strong et al., 2008), where environmental conditions are presented in detail. The dams of the test mice were CByB6F1/J, JAX stock #100009 (dams, BALB/cByJ; sires, C57BL/6J). The sires of the test mice were C3D2F1/J, JAX stock #100004 (dams, C3H/HeJ; sires, DBA/2J). In each site, breeding mice were fed LabDiet^®^ 5008 mouse chow (PMI Nutritional International, Bentwood, MO, USA). As soon as mice were weaned, they were fed LabDiet^®^ 5LG6 from the same source. Males were initially housed 3 per cage, while females were housed 4 per cage; numbers declined as mice died.

Details of the methods used for health monitoring were provided previously (Harrison et al., 2009; Miller et al., 2011; Strong et al., 2008). In brief, each of the three colonies was evaluated four to twelve times each year for infectious agents. All such surveillance tests were negative for pathogens at all three sites throughout the entire study period.

### 4.2 Removal of mice from the longevity population

Mice were removed from the study because of fighting or accidental death (e.g., during chip implantation) or chip failure, or because they were used for another experimental purpose. For survival analyses, all such mice were treated as alive at the date of their removal from the protocol and lost to follow‐up thereafter. These mice were not included in calculations of median longevity. Overall, <3% of the mice were removed from the longevity populations reported here, with no significant site differences. For details, see Methods to censor mice for lifespan statistics, Supporting Information Appendix S1 **.**

### 4.3 Estimation of age at death (lifespan)

At UM and UT, mice were examined daily for signs of ill health from the time they were set up in the experiment. At TJL, once mice were marked as ill, they were examined daily for signs of ill health. Mice were euthanized for humane reasons if so severely moribund that they were considered, by an experienced technician, unlikely to survive for more than an additional 48 hr. The TJL definitive endpoint criterion is the nonresponsiveness of a mouse to being touched, and which is usually accompanied by one or more of the following: slow respiration, feeling cold to the touch, a hunched‐up appearance with matted fur, and signs of sudden weight loss, failure to eat and drink, prominent appearing ribs and spine, and sunken hips.

The age at which a moribund mouse was euthanized was taken as the best available estimate of its natural lifespan. Mice found dead were also noted at each daily inspection.

### 4.4 Control and experimental diets

Studies with diets done at similar times are reported here, as even the diets with no effects act as useful controls. TestDiet^®^, Inc., a division of Purina Mills (Richmond, IN, USA), prepared batches of LabDiet^® ^5LG6 food containing each test substance, as well as control diets, at intervals of approximately 4 months, and shipped each batch of food to the three test sites.

1. ACA or Acarbose was purchased from Spectrum Chemical Mfg. Corp., Gardena, CA, product # A3965, CAS # 56180‐94‐0. It was fed continuously at a concentration of 400, 1,000, or 2,500 mg of ACA per kilogram of diet (ppm) to a test group of mice from cohort 2013 starting at 8 months of age. In independent groups of mice, it was fed at 1,000 mg/kg starting at 4 or 8 months of age, as indicated. Probably amounts of ACA were about a third of expected in the diet, as average concentration of acarbose in 5 batches of food pellets was 231 ± 109 (*SD*) ppm (intended dose 1,000 ppm). A primary effect of acarbose is to reduce postprandial glucose plasma levels in humans (Ruppin et al., 1988). We observed the same result as an outcome measure in this study (Figure 5). The combination of these results indicates that the same pharmacokinetic and pharmacodynamic effects of acarbose were achieved with our study design. For details, see: Experimental diets—Acarbose, Supporting Information Appendix S1.
2. UA or Ursolic acid was obtained from Wilshire Technologies, Princeton NJ, CAS # 77‐52‐1. It was fed continuously at a concentration of 2,000 ppm to a test group of mice from cohort 2013 starting at 10 months of age.
3. HBX or 2‐(2‐hydroxyphenyl) benzothiazole was obtained from Sigma Aldrich, Inc., St Louis, MO, USA, product # H50802, CAS # 835‐64‐3. It was fed continuously at a concentration of 1 ppm to a test group of mice from cohort 2012, starting at 15 months of age. In pilot studies, diets designed to contain 1 and 10 ppm HBX had 85% and 83% of expected amounts, respectively. Serum of mice fed 1 ppm HBX for 8 weeks had serum levels of 89–252 ng/ml HBX, pilot mice fed 10 ppm HBX for 8 weeks had <50 to 316 ppm, with no significant differences in serum levels between the two groups, so 1 ppm HBX was used in the full lifespan study.
4. I767d, INT‐767 or 6α‐ethyl‐24‐nor‐5β‐cholane‐3α,7α,23‐triol‐23 sulfate sodium salt was obtained from WIL Research, Inc., Ashland OH (in 2016 it was renamed Charles River Laboratories, Ashland), and the material was a proprietary product. It was fed continuously at a concentration of 180 ppm to a test group of mice from cohort 2012 starting at 10 months of age. In pilot studies, the amount of INT‐767 in the diet was 73% of the 180 ppm expected. After 8 weeks of diet containing INT‐767, amounts in serum ranged from 254 to 869 ng/ml while control serum contained less than 1.5 ng/ml.

### 4.5 Measuring amounts of interventions

Assays for acarbose, HBX, and I767 were done at UT. The initial drug material, food pellets, and mouse serum were sampled, prepared, and assayed as detailed in the material published here on line. Samples were quantified using HPLC with ultra violet detection. For details, see: Measuring amounts of Interventions**, **Supporting Information Appendix S1 **.**

### 4.6 Measures of body composition

At TJL, a Nuclear Magnetic Resonance (NMR) Imaging Instrument (EchoMRI™ 3‐in‐1, EchoMRI LLC, Houston, TX, USA) was used to measure body composition of fat, lean, free water, and total water masses. Subjects were placed in a restrainer tube (nonanesthetized), and three consecutive scans, 2 min in duration, were taken sequentially, and data were averaged for each subject. The fat calculation is measured as total body fat inclusive of organ and tissue fat and fatty acids. Lean mass is muscle plus all organs. At UT, mice were placed in a plastic cylinder (4.7 cm ID, 0.15 cm thick) in a qMRI machine (EchoMRI, Echo Medical Systems) and scanned once for 2 min. Data at both sites are expressed as % of body mass.

### 4.7 Pathology

Using standard microhistopathological methods, J. Erby Wilkinson, a board‐certified veterinary pathologist, examined 22‐ to 24‐month‐old UM‐HET3 mice: 57 females and 54 males had been fed a 1,000 ppm ACA diet since they were 4 months old. There were 14 female and 11 male controls set up at the same time, plus two previous groups of age‐matched UM‐HET3 controls, for a total of 43 females and 41 males not fed ACA. Evaluation of the ACA mice and the new set of age‐matched controls was conducted in a blinded fashion, and the data from the old control mice combined with prior records compiled by Dr. Wilkinson on earlier cohorts of untreated UM‐HET3 mice for statistical purposes.

### 4.8 Refeeding effects on blood glucose

Ten HET3 mice in each group were given one of four diets for 6 weeks starting at 4 months of age; the diets contained 2,500 ppm, 1,000 ppm, 400 ppm, or 0 ppm of ACA. Mice were then fasted from 18:00 to 09:00 the next morning. Tail vein blood was sampled (around 50 microliters) at “0” minutes before returning food, and again 30, 60, 180, and 360 min after the food was returned. Amounts of glucose were measured in each blood sample using a blood glucose meter One Touch Ultra Blue (Life‐Scan, Milpitas, CA, USA).

### 4.9 Rotarod

Rotarod performance was tested using the Rotamex‐5 (Columbus Instruments, Columbus, OH, USA) for 6 consecutive days by a technician who was blinded to the treatment groups. Testing on each day consisted of eight trials with a 10‐min rest between trials 4 and 5. Each trial began with the rotarod set at an initial rate of 4 rpm, accelerating to a maximum 40 rpm within 300 s. The latency to fall was recorded by the Rotamex‐5 software, and the average latency to fall was calculated for each day.

### 4.10 Statistical methods

Significance tests about survival effects are based upon the two‐tailed log‐rank test at *p* < 0.05, stratified by test site, with censored mice included up until their date of removal from the longevity population. Data from male and female mice are considered separately. In statistical tests described in the text,* p* values are two‐tailed and reported without adjustment for multiple comparisons, except in Figure 6. Statistical claims related to maximum lifespan are based on Wang et al., (2004), using the Fisher's exact test to compare the proportions of surviving mice, in control and test groups, at the age corresponding to the 90th percentile for survival in the joint distribution of the control and test groups. For the pooled data sets, surviving mice were enumerated at the 90th percentile age for each site separately, and these counts were combined for the overall Fisher's exact test.

### 4.11 Figure 6 statistics

For statistical analysis of the rotarod performance, the average of time‐to‐fall sessions for each animal on each day (1–6) was considered as a repeated measure in a mixed‐effect linear model with a random intercept for males and females separately and estimated the main effects of day, group (Old Control, Old ACA, and Young control), and the group‐by‐day interaction. We compared the pairwise differences between the three groups and adjusted for multiple testing using Tukey's Honest Significant difference method (HSD). For details, see Statistical methods—Figure 6 statistics Supporting Information Appendix S1.

## AUTHOR CONTRIBUTIONS

DEH, RS, and RAM are the principal investigators at the three collaborating institutions and are responsible for project design, supervision of technical personnel, interpretation of results, and preparation of manuscript drafts. JFN, KF, and SJSR provided advice on experimental design and interpretation, and comments on the manuscript. Lab manager CMA provided advice and supervised laboratory procedures and data collection at The Jackson Laboratory site, as well as organizing diet preparations for all three sites. TS did some of the statistical lifespan analyses. SJSR did physiological assays at TJL. GM provided data and analysis for Figure 3. JALG did statistical analysis of rotarod performance and advised on statistical analysis of postprandial glucose and body composition at UT. FM served as the project officer for the National Institute on Aging and contributed to program development, experimental design and analysis. EF supervised laboratory personnel and data collection at the UTHSCSA site. JEW conducted the pathology analysis. MAJ supervised assays at UT. TJS and JD proposed UA; ML proposed INT‐767; and SA and GJL proposed HBX.
