# Supplementary material for: Acarbose improves health and lifespan in aging HET3 mice
Source: Aging Cell. 2019 Jan 27;18(2):e12898. doi: 10.1111/acel.12898 (PMC6413665; doi:10.1111/acel.12898)
Supplement: Supplementary file 1 [file ACEL-18-e12898-s001.doc]

**Supporting Information: Details of Experimental Procedures Online Supplement**

Methods to censor mice for lifespan statistics

Each mouse originally entered into the study was, at the time of analysis, considered to be in one of two categories: either dead (from natural causes) or censored. Mice were censored at the age when they were no longer subjected to the mortality risks typical of un-manipulated mice. In some cases, this was because the mouse was removed because of fighting; in other cases, mice died as the result of an accident (e.g., death when anesthetized for implantation of a radio-emitting chip). In still other cases, mice were considered censored on the day in which they received an experimental treatment (such as blood sampling or tests of immune response) to which the control mice were not exposed. Kaplan–Meier analysis and log-rank comparisons among groups considered censored mice to be lost from follow-up on the day at which they were removed from the longevity protocol. No mice remained alive in any of the groups at the time of the analyses reported here.

Experimental diets - Acarbose

Assuming that a mouse eats 4 g of food each day, it consumed an average of 0.924 mg of acarbose per day. If the average mouse in our study weighed 30 g, the average dose of acarbose in was 30.8 mg acarbose/kg/day. The average single dose of acarbose in humans is 300 mg (three times daily; Harrower, 1996), which would be 5 mg/kg for a 60 kg human.

The principal mechanism of action of acarbose is the inhibition of α-glucosidase and the site of action is in the intestine (Ruppin, 1988). Previous reports indicate that less than 1% of oral acarbose is absorbed from the intestine into the blood and that acarbose is neither degraded nor absorbed by the intestine to a major degree in diabetic humans (Ruppin, 1988). A mean plasma concentration of 97 ng/mL was reported after a single dose of 300 mg to 10 healthy humans and 18.4 ng/mL after 3 months of 300 mg of acarbose 3 times daily (Harrower, 1996; Keen, 1993). In the current study, mice receiving the medium dose (1000 ppm ACA), had mean plasma levels among females and males mice of 9.66 ± 4.69 (SD) ng/mL (N = 3) and 29.9 ± 13.6 (SD) ng/mL (N = 4), respectively.

Measuring amounts of Interventions

Acarbose analytical standard was purchased from Sigma Chemical Company (St. Louis, MO, USA). HPLC grade methanol was purchased from Fisher (Fair Lawn, NJ, USA). All other reagents were were HPLC grade and purchased from Sigma Chemical Company (St. Louis, MO, USA). Milli-Q water was used for preparation of all solutions. Acarbose super stock solutions were prepared in methanol at a concentration of 1 mg/mL and stored in aliquots at –80^o^C. A working stock solution was prepared each day from the super stock solutions at a concentration of 10 μg/ml and used to spike the calibrators.

The HPLC system consisted of a Shimadzu SIL 20A HT autosampler, LC-20AD pumps (2), and an AB Sciex API 3200 tandem mass spectrometer with turbo ion spray. The LC analytical column was a Grace Alltima C18 (4.6 x 150 mm, 5 micron) purchased from Alltech (Deerfield, IL, USA) and was maintained at 25^o^C during the chromatographic runs using a Shimadzu CT-20A column oven. Mobile phase A was 10 mM ammonium formate and 0.1% formic acid dissolved in methanol:water at a ratio of 90:10. Mobile phase B was 10 mM ammonium formate and 0.1% formic acid dissolved in 100% HPLC grade methanol. The flow rate was 0.4 ml/min. Acarbose was eluted using a gradient: 0-0.1 min, 0% B; 0.1 to 4 min, 0% to 100% B; 4 to 5 min, 100% B; 5 to 5.1 min, 100% to 0% B; 5.1 to 10 min, 0% B. The Acarbose transition was 646.03 to 304.20 Da. Acarbose was quantified using the 304.2 Da fragment and the final concentration was expressed in ng/mL

*Measurement of Acarbose in Mouse Serum.* Calibrator samples were prepared daily by spiking serum and serially diluting to achieve final concentrations of 0, 2.5, 5.0, 10.0, 20.0 ng/ml. Briefly, 50 µL of calibrator and experimental serum samples were mixed with 200 µL of mobile phase B. The samples were vortexed vigorously for 2 min and then centrifuged at 13,000 *g* for 5 min at 23^o^C. Supernatants were transferred to 1.5 ml microfilterfuge tubes and centrifuged as before, and then 20 µL of the final extracts were injected into the LC/MS/MS. The peak area response of acarbose for each experimental sample was compared against a linear regression of calibrator response peak areas to quantify acarbose. The concentration of Acarbose was expressed as ng/mL.

*Measurement of Acarbose in Food Pellets.* Food calibrator samples were prepared by spiking 20 mg of pulverized food pellets with concentrations of 0, 100, 500, 1000, 2000 ng/mg. Twenty mg of calibrator and unknown samples were mixed with 4 mL of mobile phase B, vortexing vigorously, and then shaken for 10 min. Then, 300 µL of the mixture were transferred to microfilterfuge tubes and centrifuged at 13,000 *g* for 5 min and transferred to autosampler vials from which 10 µL were injected into the LC/MS/MS. The peak area response of acarbose for each experimental sample was compared against a linear regression of calibrator response peak areas to quantify Acarbose. The concentration of Acarbose was expressed as ng/mg food.

INT-767 was purchased from WIL Research, Inc. HPLC grade methanol was purchased from Fisher (Fair Lawn, NJ). All other reagents were HPLC grade and were purchased from Sigma Chemical Company (St. Louis, MO, USA). Milli-Q water was used for preparation of all solutions. INT-767 super stock solutions were prepared in methanol at a concentration of 1 mg/ml and stored in aliquots at –80^o^C. A working stock solution was prepared each day from the super stock solutions at a concentration of 10 μg/mL and used to spike the calibrators.

The HPLC system consisted of a Shimadzu SIL 20A HT autosampler, LC-20AD pumps (2), and an AB Sciex API 3200 tandem mass spectrometer with turbo ion spray. The LC analytical column was a Grace Alltima C18 (4.6 x 150 mm, 5 micron), purchased from Alltech (Deerfield, IL), and maintained at 60^o^C during the chromatographic runs using a Shimadzu CT-20A column oven. Mobile phase A was 10 mM ammonium formate and 0.1% formic acid dissolved in methanol:water at a ratio of 90:10. Mobile phase B was 10 mM ammonium formate and 0.1% formic acid dissolved in 100% HPLC grade methanol. The flow rate of the mobile phase was 0.5 ml/min. INT-767 was eluted from the analytical column with a step gradient: 0 to 2.1 min, 0% B; 2.1 to 8.1 min, 100% B; 8.1 to 15 min, 0% B. INT-767 was quantified using the parent ion, 471.2 Da and the final concentration of INT-767 was expressed in ng/mL.

*Measurement of INT-767 in Mouse Serum.* Serum calibrator samples were prepared daily by spiking serum and serially diluting to achieve final concentrations of 0, 1.5, 3.13, 6.25, 12.5, 50 and 100 ng/ml. Briefly, 100 µL of calibrator and experimental serum samples were mixed with 200 µL of mobile phase B, vortexed vigorously for 5 min, and then centrifuged at 13,000 *g* for 5 min at 23^o^C. Supernatants were transferred to 1.5 mL microfilterfuge tubes and centrifuged as above, and then 10 µL of the final extracts were injected into the HPLC/MS/MS system. The concentration of INT-767 was expressed as ng/mL

*Measurement of INT-767 in Food Pellets.* Calibrator samples were prepared by spiking pulverized 100 mg of food pellet samples at concentrations of 0, 10, 25, 50, 100, 200 ng/mg. INT-767 was quantified in mouse food by mixing 100 mg of calibrator and experimental samples with 4 mL of mobile phase B, vortexing the samples vigorously and shaking them for 10 minutes. Then, 300 µL of the solution were transferred to microfilterfuge tubes and centrifuged at 13,000 *g* for 5 min. The samples were transferred to autosampler vials from which 10 µL of the final samples were injected into the HPLC/MS/MS system. The peak area response of INT-767 for each experimental sample was compared against a linear regression of calibrator response peak areas to quantify INT-767. The concentration of INT-767 was expressed as ng/mg food.

HBX and diazepam were purchased from Sigma Aldrich (St. Louis, MO, USA). HPLC grade methanol and acetonitrile were purchased from Fisher (Fair Lawn, NJ, USA). All other reagents were HPLC grade and were purchased from Sigma Chemical Company (St. Louis, MO, USA). Milli-Q water was used for preparation of all solutions. HBX and super stock solutions were prepared in methanol at a concentration of 1 mg/ml and stored in aliquots at –80^o^C. Working stock solutions (10 and 100 μg/mL) were prepared fresh each day from the super stock solutions and used to spike the calibrator samples for both ground pellets and blank plasma. Diazepam internal standard was prepared in methanol at a concentration of 10 µg/ml and stored at –80^o^C.

The HPLC system was a Waters 600 Pump Controller, Waters 717 Plus autosampler, and a Waters 2487 dual wavelength absorbance detector (λ 1 = 214 nm; λ 2 = 235 nm). The analytical column was a Grace Alltima C18 (4.6 x 150 mm, 5 µ) purchased from Alltech (Deerfield, IL, USA) and maintained at 50^o^C during the chromatographic runs using a Brinkmann TC-50 column oven. The isocratic mobile phase was 50% Acetonitrile, 50% 12 mM K_2_HPO_4_, pH 2.5. The flow rate of the mobile phase was 1.0 mL/min. HBX eluted from the HPLC column at 28.2 min, the internal standard diazepam at 5.6 min.

*Quantification of HBX in Plasma.* Calibrator samples were prepared by spiking blank plasma with 0, 10, 50, 100, 500, and 1000 ng/mL. Briefly, tubes containing 100 µL of plasma calibrator and experimental samples were mixed with 10 µL of 10 µg/mL diazepam (internal standard) and 500 µL of mobile phase. The samples were vortexed vigorously for 5 min, shaken for 30 min, and then centrifuged at 3,200 *g* for 30 min at 23^o^C. Supernatants were transferred to microcentrifuge tubes and centrifuged at 3,200 *g* for 20 min. 100 µl of the final samples were injected into the HPLC/MS/MS system. The ratio of the peak areas of HBX to that of the internal standard diazepam (response ratio) for each experimental sample was compared against a linear regression of calibrator response ratios to quantify HBX. The concentration of HBX was expressed as ng/mL.

*Measurement of HBX in Food Pellets.* Calibrator samples were prepared by spiking pulverized 100 mg of food pellet samples with HBX concentrations of 0, 10, 25, 50, 100, 200 ng/mg. Briefly, 100 mg of calibrator and pulverized experimental food pellet samples were mixed with 10 µL of 10 µg/mL diazepam (internal standard) and 500 µL of mobile phase (pH 6.7) solution. The samples were vortexed vigorously for 1 min, shaken for 30 min, and then centrifuged at 3,200 *g* for 20 min at 23**^o^**C. Supernatants were transferred to microcentrifuge tubes and centrifuged at 3,200 *g* for 10 min. 100 µl of the final samples were injected into the HPLC/MS/MS system. The ratio of the peak areas of HBX to that of the internal standard diazepam (response ratio) for each experimental sample was compared against a linear regression of calibrator response ratios to quantify HBX. The concentrations of HBX in food pellets were expressed as ng/mg.

Statistical methods - Figure 6 statistics

The statistical significance of the group-by-day interaction would indicate that the groups have difference learning trajectories (as distinct from just being higher or lower on average). If the interaction term was not significant (P > 0.05), then the interaction was removed from the model. In order to reduce the number of comparisons, day was considered as a linear or a quadratic effect, depending on the significance of the quadratic term. We further compared each of the three subgroups to one another by excluding the other group for both males and females (Old Control vs. Young; Old Control vs. Old ACA; Young vs. Old ACA). In these models, we only considered the main effects of day (1–6) and group. Pairwise differences between the three groups were compared and adjusted for multiple testing using Tukey’s Honest Significant difference method (HSD). All tests were two-sided with significance threshold of P < 0.05.

P values for differences in performance between ACA treated and matched age controls:

On day 6 - females = 0.0001, males = 0.48.

Average over days 1-6 – females = 0.01, males = 0.33.

Rate of learning (slope over days 1-6) – females = 0.009, males =0.33.

P values for differences in performance between young and old controls:

On day 6 - females = 0.02, males = 0.06.

Average over days 1-6 – females = 0.006, males = 0.007.

Rate of learning (slope over days 1-6) – females = 0.01, males =0.6.

We used R (v3.1+, Vienna, Austria) and the lmer1 and lmerTest 2 packages.
